# Comprehensive illustration of transcriptomic and proteomic dataset for mitigation of arsenic toxicity in rice (*Oryza sativa* L.) by microbial consortium

**DOI:** 10.1016/j.dib.2022.108377

**Published:** 2022-06-10

**Authors:** Surabhi Awasthi, Reshu Chauhan, Yuvraj Indoliya, Abhishek Singh Chauhan, Shashank Kumar Mishra, Lalit Agrawal, Sanjay Dwivedi, Shiv Naresh Singh, Suchi Srivastava, Poonam C. Singh, Puneet Singh Chauhan, Debasis Chakrabarty, Sudhakar Srivastava, Rudra Deo Tripathi

**Affiliations:** a CSIR – National Botanical Research Institute, Council of Scientific and Industrial Research, Rana Pratap Marg, Lucknow, 226 001, India; b Plant Stress Biology Laboratory, Institute of Environment and Sustainable Development, Banaras Hindu University, Varanasi, 221005, India; c Department of Agriculture and Allied Science, Doon Business School, Dehradun, Uttarakhand, India; d Academy of Scientific and Innovative Research (AcSIR), Anusandhan Bhawan, 2 Rafi Marg, New Delhi, 110 001, India

**Keywords:** Antioxidant enzymes, *Chlorella vulgaris, Pseudomonas putida*, Phytohormone, Transporters

## Abstract

The present article represents the data for analysis of microbial consortium (*P.putida*+*C.vulgaris*) mediated amelioration of arsenic toxicity in rice plant. In the current study the transcriptome profiling of treated rice root and shoot was performed by illumina sequencing (Platform 2000). To process the reads and to analyse differential gene expression, Fastxtoolkit, NGSQCtoolkit, Bowtie 2 (version 2.1.0), Tophat program (version 2.0.8), Cufflinks and Cuffdiff programs were used. For Proteome profiling, total soluble proteins in shoot of rice plant among different treatments were extracted and separated by 2D poly acrylamide gel electrophoresis (PAGE) and then proteins were identified with the help of MALDI-TOF/TOF. In gel based method of protein identification, the isoelectric focusing machine (IPGphor system,Bio-Rad USA), gel unit (SDS-PAGE) and MALDI-TOF/TOF (4800 proteomic analyzer Applied Biosystem, USA) were used for successful separation and positive identification of proteins. To check the differential abundance of proteins among different treatments, PDQuest software was used for data analysis. For protein identification, Mascot search engine (http://www.matrixscience.com) using NCBIprot/SwissProt databases of rice was used. The analyzed data inferred comprehensive picture of key genes and their respective proteins involved in microbial consortium mediated improved plant growth and amelioration of As induced phyto-toxicity in rice. For the more comprehensive information of data, the related full-length article entitled “Microbial consortium mediated growth promotion and Arsenic reduction in Rice: An integrated transcriptome and proteome profiling” may be accessed.

## Specifications Table

**Table:** 

Subject	Agriculture science Environmental Microbiology
Specific subject area	Mitigation of arsenic toxicity in rice
Type of data	Figure Table Excel sheet
How data were acquired	The rice plants were grown hydroponically for 10 days (d) and exposed to different treatments *viz.,* control, *Pseudomonas putida* (*Pp*) and *Chlorella vulgaris* (*Cv*) consortium, As(V) (50 μM; Na_2_HAsO_4_.7H_2_O; Sigma), and As+*Pp*+*Cv* for 15 d. The structural anomalies of rice roots were examined under the light microscope (Nikon, ECL, IPSETE 300) and photographed with the help of digital imaging system (Nikon DXM1200S (Fig 1.2). For illumina sequencing, Fastxtoolkit, NGSQCtoolkit, Bowtie 2 (version 2.1.0), Tophat program (version 2.0.8), cufflinks package and cuffdiff program (version 2.0.2) were used (Table 1). MeV software was used to generate the heat maps (Fig. 2). Venn 2 software was used to produce venn diagrams (Fig. 3). The higher-level match set protein master gel was generated with the help of PDQuest software [version 8.0.1, Bio-Rad (Fig. 5).
Data format	Raw Analyzed
Description of data collection	Hydroponically grown rice seedlings were exposed to different treatments *viz.,* control, *Pp* and *Cv* consortium, As(V) (50 μM; Na_2_HAsO_4_.7H_2_O; Sigma) and As+*Pp*+*Cv* consortium. After 15 d of treatment, the plants were harvested and stored in liquid nitrogen for for transcriptome and proteome profiling. Transcriptome profiling of rice plant was carried out using Illumina Sequencing (Illumina HiSeq 2000 platform) to generate 100 bp end reads which were further processed for gene expression analysis. For proteome analysis total soluble protein was extracted by phenol extraction method and differentially expressed proteins were identified by MALDI-TOF/TOF.
Data source location	The experiment was carried out in National Botanical Research Institute, Lucknow, India.
Data accessibility	The sequencing reads of all root and shoot samples have been submitted to NCBI BIOPROJECT (PRJNA648294). The data can be accessed with the help of below given link. With the article Repository name: NCBI BIOPROJECT Data identification number (permanent identifier, i.e., DOI number): PRJNA648294 https://doi.org/10.1016/j.ecoenv.2021.113004 Direct URL to data: https://www.ncbi.nlm.nih.gov/bioproject/?term=PRJNA648294 and ENA Browser (ebi.ac.uk).
Related research article	S. Awasthi, R. Chauhan, Y. Indoliya, A. S. Chauhan, S. K. Mishra, L. Agrawal, S. Dwivedi, S.N. Singh, S. Srivastava, P. C. Singh, P. S. Chauhan, D. Chakrabarty, S. Srivastava, R. D. Tripathi, Microbial consortium mediated growth promotion and Arsenic reduction in Rice: An integrated transcriptome and proteome profiling, Ecotox. Environ.Safety. Doi: https://doi.org/10.1016/j.ecoenv.2021.113004

## Value of the Data

• The collective analysis of genes and proteins provides deeper insights into the metabolic networks of arsenic-plant-microbe interaction for mitigation of arsenic toxicity in rice plant.
• The present analysis would be helpful for the scientific community working in the field of crop contamination of heavy metals.
• The detailed analysis of expression of genes and proteins would be helpful for the development of low grain arsenic rice variety that is urgently required. The present research would help in gaining deeper insights into the microbial interactions with rice plants under the arsenic stressed conditions. This would allow selection of more potential bacteria and alga for designing consortia for achieving more fruitful results.
• The large database generated, pertaining to arsenic stress responsive-, microbial consortia responsive- and arsenic+microbial consortia-responsive genes and proteins would help to understand the role of various genes and proteins in arsenic stress tolerance.
• The study would also be beneficial in taking microbial consortia to the field, for reducing arsenic in rice grains in arsenic contaminated fields.

## 1 Data Description

**Fig. 1.1:**
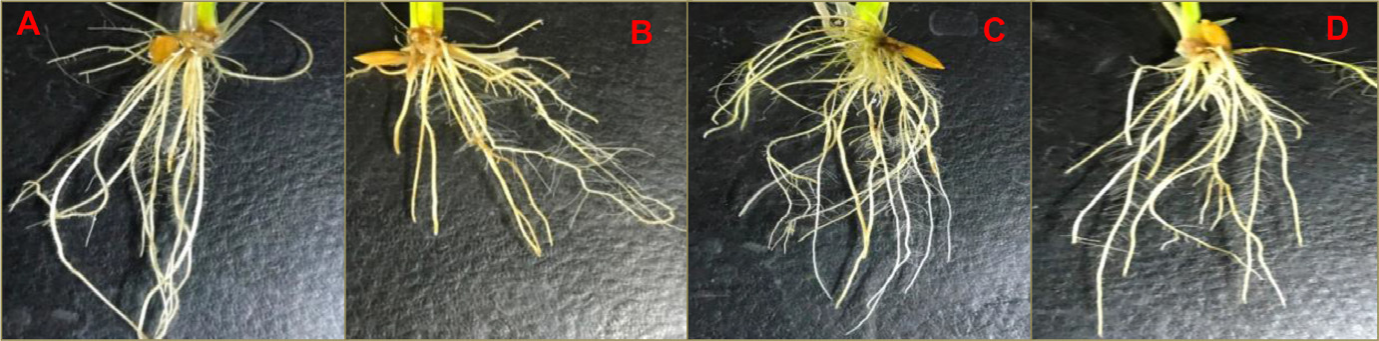
Microbial consortium mediated amelioration of arsenic toxicity in rice. Changes in growth of rice root and root hairs were monitored after 15 d in Control (A); As (B), *P.putida+C.vulgaris* (C) and As+*P.putida+C.vulgaris* (D). Five plants were used for analysis and representative images are presented (n=5).

**Fig. 1.2:**
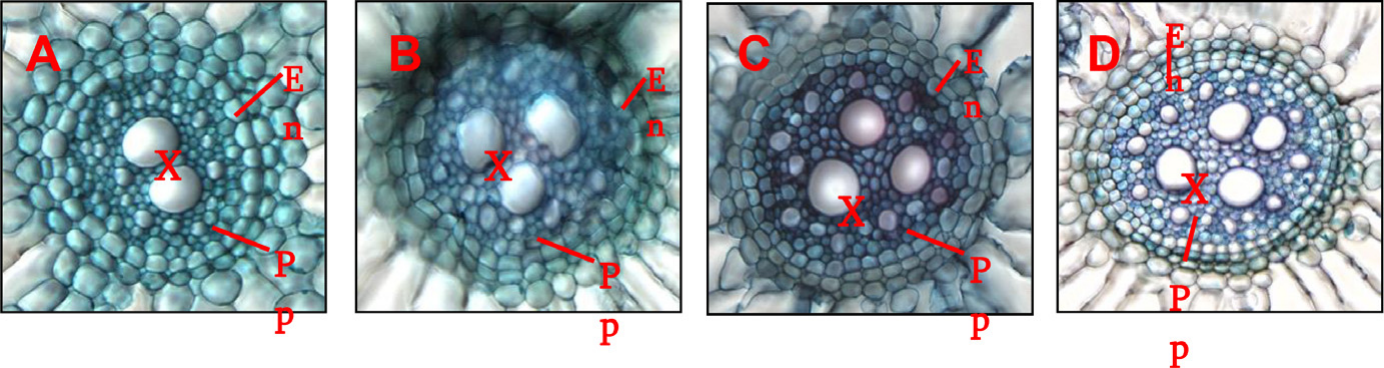
Changes in anatomical structure (epidermal layers) were monitored in transverse sections of rice roots after 15d in control (A); As (B), *P.putida+C.vulgaris* (C) and As+*P.putida+C.vulgaris* (D). Panels depict that endodermis, xylem and pith parenchyma cells were distorted during As exposure (B) but significantly improved during microbial consortium supplementation in the presence of As (D). Symbols denote: En- endodermis; X - xylem; Pp – Pith parenchymatous cells. Pictures were taken at 20X magnification using EVOS digital inverted microscope and were enlarged in equal proportion.

Arsenic (As) is most toxic environmental pollutants and hinders plant growth as indicated by reduction in root hair and As induced structural anomalies. The supplementation of microbial consortium improved the plant growth in terms of improved growth of root hair (Fig. 1.1) and maintains the cellular integrity by alleviating the As induced cellular damage and toxicity (Fig. 1.2).

Transcriptomic and proteome analysis in rice seedlings under As stress identified several As responsive genes including transcription factors, phytohormones and As transporters as well as their respective proteins was carried out. Several genes were found to be differentially expressed in root and shoot of rice plant among different treatments. The data of RNA sequencing performed in root and shoot of rice plant in different treatments *viz.,* Control, As, *P.putida+C.vulgaris* and As+*P.putida+C.vulgaris* is presented in Table 1.

**Table 1:** Summary of fastq file generated from RNA sequencing showing average statistics of all samples individually.

	SHOOT				ROOT			
File Name	Control	As	*P. putida + C. vulgaris*	As+ *P. putida + C. vulgaris*	Control	As	*P. putida + C. vulgaris*	As+ *P. putida + C. vulgaris*
Total number of reads	24721794	24429514	21520424	22348793	25349956	26837996	24458041	24063156
Total number of HQ reads	23848896	23573973	20842745	21575566	24111248	25951739	23682044	23286480
Percentage of HQ reads	96.47%	96.5%	96.85%	96.54%	95.11%	96.7%	96.83%	96.77%
Total number of bases	1878856344	1856643064	1635552224	1698508268	1926596656	2039687696	1858811116	1828799856
Total number of bases in HQ reads	1812516096	1791621948	1584048620	1639743016	1832454848	1972332164	1799835344	1769772480
Total number of HQ bases in HQ reads	1779729223	1760374439	1557944730	1612238896	1797871672	1938256344	1769987054	1741295266
Percentage of HQ bases in HQ reads	98.19%	98.26%	98.35%	98.32%	98.11%	98.27%	98.34%	98.39%
Total number of HQ filtered reads	23848896	23573973	20842745	21575566	24111248	25951739	23682044	23286480
Percentage of HQ filtered reads	96.47%	96.5%	96.85%	96.54%	95.11%	96.7%	96.83%	96.77%

**Fig. 2:**
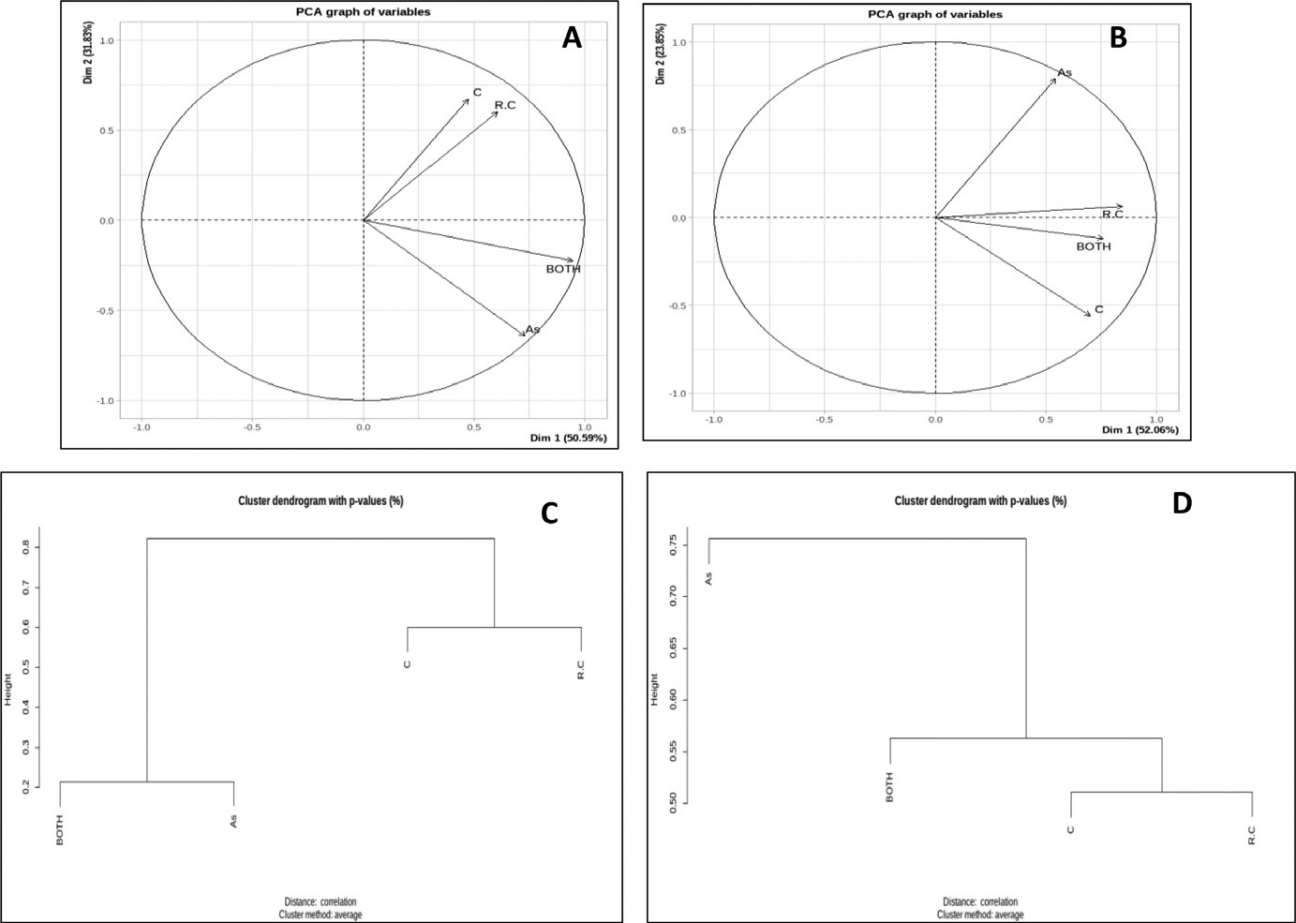
Global expression pattern analysis of genes among different treatments *viz*., control, *P.putida*+*C.vulgaris*, Arsenic and As+*P.putida*+*C.vulgaris* by using the threshold of *P*≤ 0.5 and |log2 Fold Change| > ±1.5. Figure A and B are showing PCA plot in root and shoot respectively, whereas C (root) and D (shoot) represent hierarchical clustering algorithm. This figure represents overall diverse expression pattern of genes among different treatments in both root and shoot. In root, control and *P.putida+C.vulgaris* treated plants were in similar clade and showed least variation, whereas, As and As+ *P.putida+C.vulgaris* were grouped together (Fig. 2A, Fig. 2C). In shoot, control, *P.putida+C.vulgaris* alone and As+ *P.putida+C.vulgaris* treated plants showed least variation in gene expression compared to As alone exposure that lied in different clade due to distinct expression patterns (Fig. 2B, Fig. 2D).

To compare the pattern genes expression observed in all three treatments (As, *P.putida+C.vulgaris* and As+*P.putida+C.vulgaris*) in comparison to control, hierarchical clustering and PCA was performed in both rice root and shoot (Fig. 2 A-D).

**Fig. 3:**
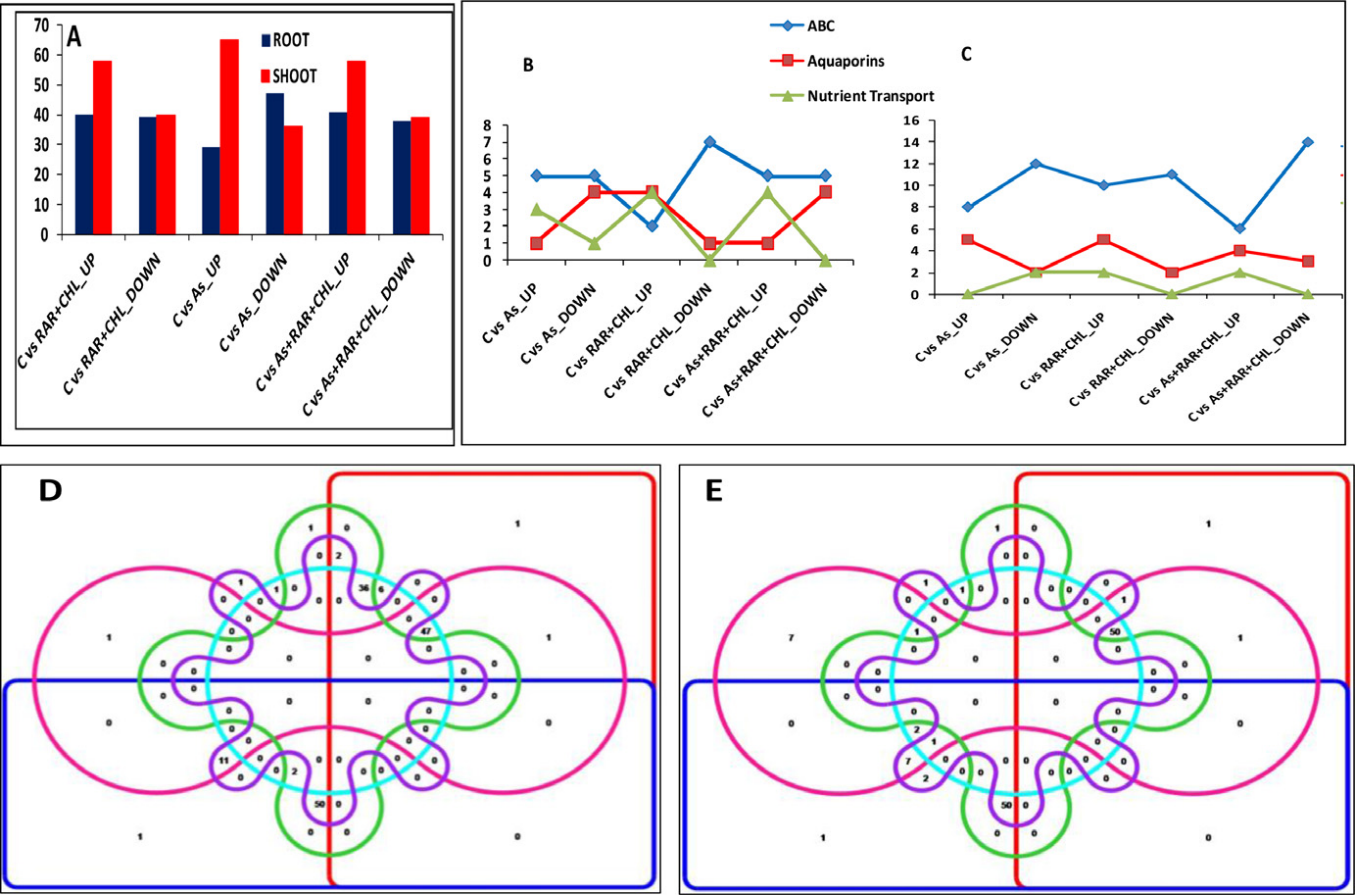
Changes in expression pattern of genes belonging to transport and transcription factor families in As, *P.putida*+*C.vulgaris*, and As+*P.putida*+*C.vulgaris* treatments as compared to control. Figure 3A is showing total number of differentially expressed genes of transporters in root and shoot. Figure 3B & 3C are showing number of up- and down-regulated ABC, aquaporin and nutrient transporters genes among different treatments compared to control in both root and shoot respectively. Figure 3D (root) and 3E (shoot) represents venn diagram showing number of unique and common up and down-regulated genes of transcription factors families among all treatments (As, *P.putida*+*C.vulgaris*, and As+*P.putida*+*C.vulgaris*) compared to control. In venn diagram red box showing number of up-regulated genes in *P.putida+C.vulgaris* treated plants, blue box showing number of down-regulated genes in *P.putida*+*C.vulgaris* treated plants, pink and green box showing number of up- and down-regulated genes respectively, in As+*P.putida*+*C.vulgaris* exposed plants. Sky blue box showing number of up- regulated genes in Arsenic alone treatment whereas purple box indicates number of down- regulated genes in arsenic treatment (Fig. 3D, 3E). In Figure 3D, 94 genes belong to transcription factor family were found differentially up-regulated and 65 down-regulated genes in *P.putida*+*C.vulgaris* treatment compared to control, whereas, 51 genes were up- and 92 genes were down-regulated in As+*P.putida*+*C.vulgaris*. 93 genes were differentially up-regulated and 65 were down-regulated in As alone treatment. In shoot (Fig. 3E), the number of up-and down-regulated genes in *P.putida*+*C.vulgaris* were 54 and 64 respectively. 77 genes were found differentially up-regulated in As+*P.putida*+*C.vulgaris* and 52 genes were down-regulated compared to control. In arsenic alone treatment 56 genes were up-regulated whereas, 63 genes were down-regulated. X axis of Figure 3A, 3B & 3C represents the different treatments compared to control and Y axis represents the total number of genes.

Transporters are the major effectors regulating the uptake, accumulation and transport of As in plants [1]. Nutrient elements also play vital role in As tolerance. Thus modulation of nutrient element transport in the presence of microbial consortium during As stress improves the plant growth and also involved in reducing As accumulation in rice plant (Fig. 3A, B & C).

Transcription factor families shows a diverse expression pattern during As, *P.putida*+*C.vulgaris*, and As+*P.putida*+*C.vulgaris* exposure compared to control. Various TFs families were found differentially expressed in all three treatments *viz.,* As, *P.putida+C.vulgaris* and As+*P.putida+C.vulgaris* compared to control. A total of 155 and 236 TF genes showed differential expression in both root and shoot, respectively in comparison to control. Venn diagram showing number of up- and down-regulated genes belong to various TFs in root and shoot respectively. (Fig. 3D & 3E).

**Fig. 4:**
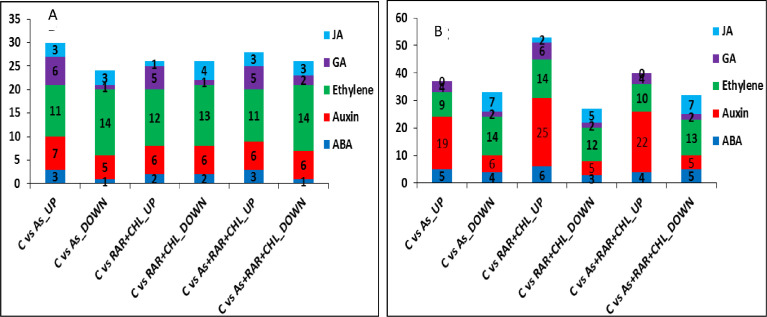
Hormonal regulation pattern and differential expression of genes involved in abiotic stress and defense responses in As, *P.putida*+*C.vulgaris*, and As+*P.putida*+*C.vulgaris* treatments as compared to control. Figure 4A and 4B are showing the number of up- and down-regulated genes belonging to harmones in root and shoot respectively. The numbers on bars represents the number of genes responsive to different hormone *viz*., ABA, auxin, ethylene, gibberellic acid and jasmonic acid respectively which were indicated by different colors. X axis represents the different treatments compared to control and Y axis represents the total number of genes.

A total of 60 phytohormone responsive genes in root and 86 genes in shoot were found differentially expressed among all three treatments compared to control. The genes belonged to ABA, auxins, ethylene and giberellin phytohormones (Fig. 4A-B).

**Fig. 5:**
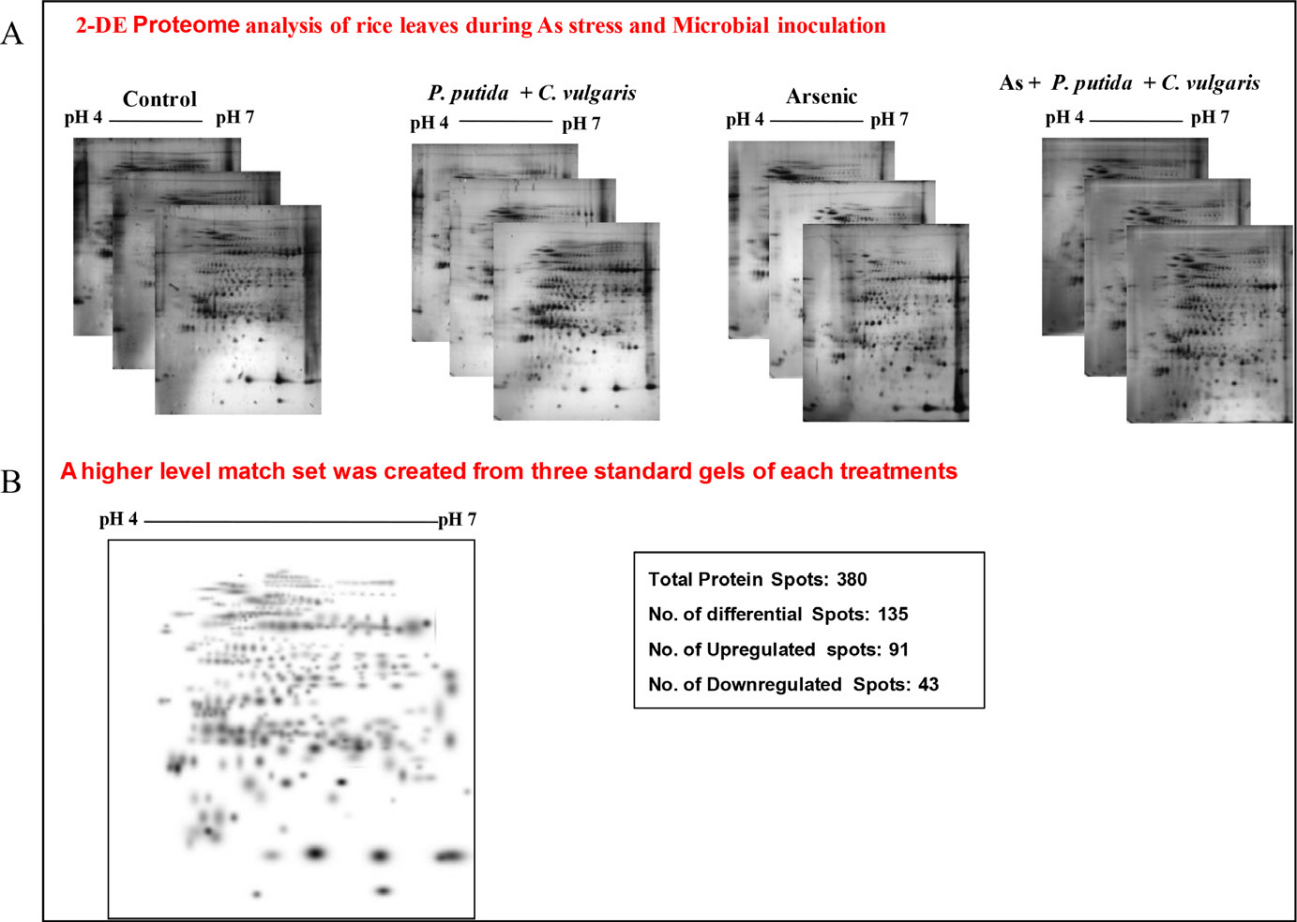
(A) Proteome profiling of rice leaves through 2-DE among all four rice treatments *viz.,* control, As, *P.putida*+*C.vulgaris* alone, and As+*P.putida*+*C.vulgaris* showing difference in protein spots in all treatments. (B) Master gel showing total proteins as well as up- and down-regulated proteins in comparison to control.

To check the abundance of differentially expressed identified proteins in all four treatments of rice plant, the SDS-PAGE and MALDI-TOF/TOF analysis was performed [2]. After extraction of total soluble protein of rice shoot, the proteins were separated firstly based on PI value and secondly considering their molecular masses. Silver stained gels showed different visualization of protein spots indicating differential abundance of proteins in rice shoot, in all four treatments. Different treatments of rice leaves showed large difference in abundance of proteins. For quantification and and accessing differential expression of proteins, PDQuest software was used. Mean of high quality spots was used to measure quantity of spots on master gel (Fig. 5). In data analysis, protein spots with increase /decrease of 2.5 fold was considered for diffrential protein expression in at least one treatment. For statistical significance among treatments, one-way ANOVA with p < 0.01value was performed (Fig. 5B). The marked protein gel pieces were processed for MALDI-TOF/TOF and seventy eight proteins were significantly identified (Fig. 5).

**Fig. 6:**
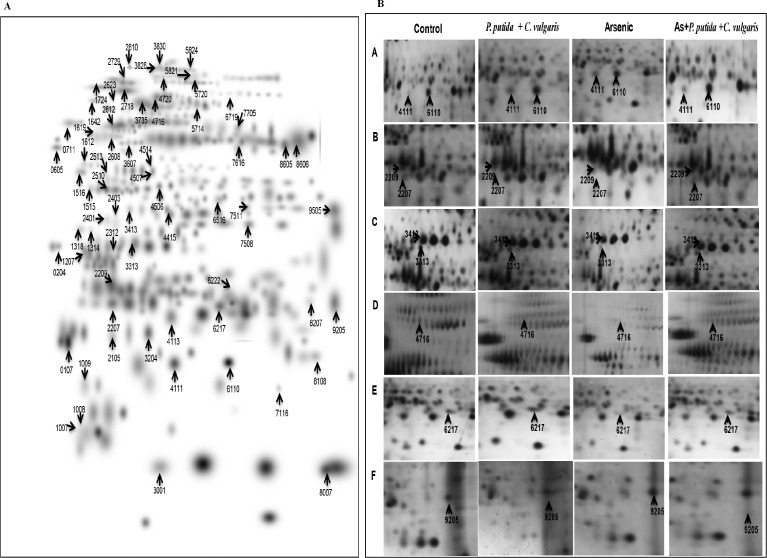
**(A).** A higher-level master image generated by using PDQuest from replicate gels of each treatment. **(B)**. The spot view of arrow represent the zoomed-in gel sections in control, As, *P.putida*+*C.vulgaris* alone, and As+*P.putida*+*C.vulgaris*. Numbers indicates the specific spot IDs.

**Fig. 7:**
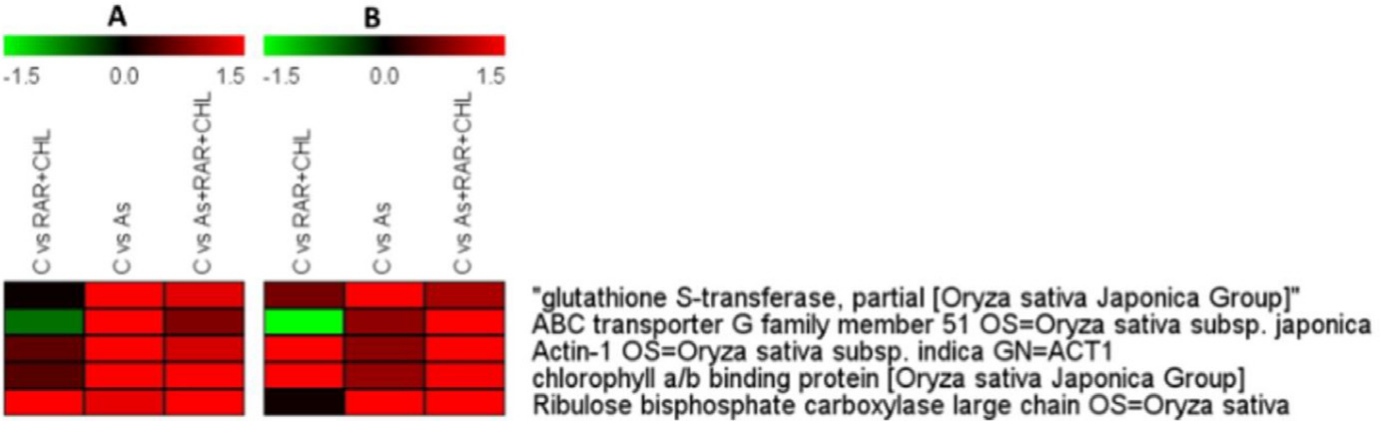
Comparative expression analysis of transcriptome and proteome analysis during As exposure and supplementation of microbial consortium (*P.putida+C.vulgaris* and As+ *P.putida+C.vulgaris*) showing similar expression dynamics among different treatments compared to control. Panel A represents proteome data and Panel B for Transcriptome data.

A master gel of higher level matchset of proteins was produced by using PD Quest software (Fig. 6). Figure 6B represents a spot view of selected identified proteins. The proteins which were identified with the help of MALDI-TOF/TOF were mainly of energy metabolism, photosynthesis, cell signalling, ROS & defense, amino acid metabolism, transport and protein synthesis. The MeV software was used to generate the heatmaps showing common genes and their respective proteins representing expression pattern among different treatments at both transcript as well as proteome level (Fig. 7).

## 2 Experimental Design, Materials and Methods

Rice seedlings (Triguna variety) were grown hydroponically in Hewitt nutrient medium for 7 days [3]. After 7 day of acclimatization the plants were inoculated with consortium of *Pseudomonas putida*, MTCC 5279 (*P.putida*) and *Chlorella vulgaris* (*C.vulgaris*) and also exposed to arsenate [50 μM As(V)]. After 15 days of treatment rice plants supplemented with consortium alone as well as As(V)+*P.putida*+*C.vulgaris* were harvested and stored in liquid nitrogen for illumina sequencing and proteome profiling (2D- gel electrophoresis and MALDI-TOF/TOF). The freshly harvested plants of different treatments were also stored in 70% ethanol to check the structural anomalies.

To analyze the structural anomalies, section of rice roots was manually cut and stained following the method [4]. Further, the stained sections were mounted with the help of DPX. The prepared slides of rice root tissues were examined under the light microscope. The sections were photographed for tissue profiling with the help of digital imaging system (Nikon DXM1200S) fixed on the top of light microscope (Nikon, ECL, IPSETE 300) (Fig 1.2).

### 2.1 Isolation of RNA for illumina sequencing and gene expression analysis

Total RNA from liquid nitrogen freezed rice root and shoot was isolated with the help of RNA isolation kit (RNeasy Plant Mini Kit, QIAGEN, MD). RNA samples from each treatment *viz*., control, As(V), *P.putida*+*C.vulgaris* and As+*P.putida*+*C.vulgaris* of rice root and shoot (independent triplicate of each sample) were pooled and used for sequencing. The total RNA from each sample having value 260/280 and RIN value 8.5 was used for sequencing. Illumina HiSeq was used for the preparation of cDNA library. Libraries were run on illumina HiSeq sequencing platform for the generation of 100bp end reads. Bowtie (version 2.0) was used for trimmed paired end reads and for the removal of adapter sequences and to improve quality followed by reference-based pair-wise alignment against reference rice genome (Ensembl (http://plants.ensembl.org/Oryza_sativa/Info/Index) [Release-26]) with the help of Tophat program (version 2.0.8) with default parameters [5]. In *Oryza sativa*. MSU 21 genome, the uniquely mapped reads were calculated [6]. Cufflinks program (version 2.0.2) was used to estimate expression of gene by calculating uniquely aligned reads and cuffdiff program (version 2.0.2) was used for differential gene expression analysis

### 2.2 Analysis of relatedness among different samples, gene annotation and K-means clustering

For the analysis of global gene expression and relatedness among different treatments of rice root and shoot, the PCA and hierarchical clustering was performed. PCA analysis was carried out with the help of factomineR and hierarchical clustering was performed by hclust software [7]. Differentially expressed genes which were involved in same pathways were annotated with the help of PageMan analysis [8]. MeV 4.9 software (http://sourceforge.net/pro-jects/mev-tm4/files/mev-tm4/) was used for K mean clustering and heat map generation. Heat maps were generated for the differentially expressed genes with p ≤ 0.05 value.

### 2.3 Extraction of total soluble proteins and identification by 2D- gel electrophoresis and MALDI-TOF/TOF

Total soluble protein was extracted by phenol extraction method from three biological replicates of each treatment *viz.,* control, As(V), *P.putida*+*C.vulgaris* and As+*P.putida*+*C.vulgaris* to normalize the variation [2]. Total shoot protein was quantified by Bradford method (Bio-Rad, USA). The first dimension of 2D gel electrophoresis was separation of proteins on the basis of their charge. In this process, the first step was rehydration of proteins. An equal amount of protein was rehydrated overnight (16 hours) passively on immobilized pH gradient (IPG) strips of 13 cm and pH ranges 4-7. The rehydrated proteins were electro focused in isoelectric focusing (IEF) rehydration buffer for the separation of proteins on the basis of their charges in IPGphor system (Bio-Rad USA). This step was completed at 20°C up to 25000 Vh and takes 8-10 hours. The focused protein strips were then treated with 1% w/v dithiothreitol (DTT) and 2.5% w/v iodoacetamide (IAA) for reduction and alkylation of proteins respectively. The second dimension was SDS-PAGE, for the separation of proteins of on the basis of mass. In SDS-PAGE the strips were placed on the top of 12.5% poly acrylamide gels and fixed with 0.5% agarose solution. For the visualization of protein spots the gels were first fixed and then stained in silver stain [silver stain plus kit (Bio-Rad, USA)].

### 2.4 PDQuest Analysis and protein identification

For the data analysis with the help of PDQuest software (version 8.0.1, Bio-Rad), the gels were scanned for image acquisition by Bio-Rad Flour S system that was equipped with camera (12-bit). Images of each biological replicate of all four treatments (control, *P.putida*+*C.vulgaris*, Arsenic and As+*P.putida*+*C.vulgaris*) [1]. The PDQuest analysis was carried out for the quantification of proteins on the basis of one of the parameters viz., molecular mass, spot quality and isoelectric point of individual protein [8]. In the PDQuest analysis the spots having reproducibility in at least two out of three replicate gels were considered.

After PDQuest analysis, the differentially expressed protein gel spots were marked, cut and digested in trypsin (Promega Corporation, MA, USA). The crushed gel pieces were dehydrated (acetonitrile: 50mM ammonium bicarbonate in 2:1 ratio) and rehydrated (25mM ammonium bicarbonate) repeatedly till the gel pieces appears white in color. The fully dried gel pieces were then rehydrated and incubated at 37 °C for 10-12 hrs. in 0.2µg/µl trypsin prepared in 50mM ammonium bicarbonate). The processed gel pieces were treated with 1% TFA prepared in 50% acetonitrile for extraction of peptides. This was followed by vacuum centrifugation to concentrate peptide solution up to 20 µl. Before spotting on MALDI plate, the peptide solution was mixed with matrix (α-cyano-4-hydroxycinnamic acid). The MALDI-TOF/TOF analysis was performed in 4800 proteomic analyzer (Applied Biosystem, USA). The mono isotopic peptide masses, collected by MALDI-TOF/TOF were then analyzed with the help of 4000 Series Explorer software version 3.5 in which in data dependent mode, the spectra were collected. NCBIprot/SwissProt databases of rice, *Oryza sativa* (172275 sequences) was used to search obtained MS/MS data using Mascot search engine ((http://www.matrixscience.com) for protein identification. The followed criteria for database search included (i) taxonomy-*Oryza sativa*, (ii) MS/MS tolerance- ±0.2 Da, (iii) peptide tolerance- ±100ppm, (iv) peptide charge- +1, (v) fixed modification-cysteine, carbamidomethylation, (vi) maximum allowed missed cleavage- 1, (vii) variable modification: methionine oxidation, (viii) instrument type: MALDI-TOF/TOF. Protein spots with MOWSE score value greater than significant threshold level were considered as positive protein identification.

## Ethics Statement

Not applicable.

## CRediT Author Statement

**Surabhi Awasthi** and **Reshu Chauhan:** performed all the experiments; **Surabhi Awasthi** and **Reshu Chauhan:** designed the experiments and analyzed the data; The manuscript was written by Surabhi Awasthi and Reshu Chauhan and reviewed by Sudhakar Srivastava, Puneet Singh Chauhan, Suchi Srivastava, Lalit Agrawal, Debasis Chakraborty and Rudra Deo Tripathi; **Shashank kumar Mishra, Yuvraj Indoliya, Abhishek Singh Chauhan** and **Shiv Naresh Singh:** helped during experiments; **Sanjay Dwivedi:** provided technical assistance; **Sudhakar Srivastava** and **Rudra Deo Tripathi:** finalized manuscript for submission.

## Declaration of Competing Interest

The authors declare that they have no known competing financial interests or personal relationships that could have appeared to influence the work reported in this paper.

## Data Availability

Combined Transcriptome and Proteome Profiling Reveals Microbial Consortium mediated Growth Promotion and Reduction of Arsenic Burden and Toxicity in Rice (Oryza sativa L.) (Reference data) (NCBI).

## Appendix Supplementary materials

Image, application 1
